# Perceived feasibility of a primary care intervention for Tobacco Cessation on Prescription targeting disadvantaged groups in Sweden: a qualitative study

**DOI:** 10.1186/s13104-016-1949-y

**Published:** 2016-03-09

**Authors:** Anne Leppänen, Olivia Biermann, Carl Johan Sundberg, Tanja Tomson

**Affiliations:** Department of Learning, Informatics, Management and Ethics, Karolinska Institutet, Tomtebodavägen 18 A, 171 77 Stockholm, Sweden; Department of Physiology and Pharmacology, Karolinska Institutet, von Eulers väg 8, 171 77 Stockholm, Sweden

**Keywords:** Tobacco use cessation, Prescriptions, Primary health care, Vulnerable populations, Sweden

## Abstract

**Background:**

There is a lack of scientific evidence on how socioeconomically disadvantaged tobacco users can be reached with tobacco cessation interventions in Swedish primary healthcare (PHC). In this setting other lifestyle interventions are available by prescription, and there is the potential to develop a similar tool for tobacco cessation. The aim of this study was thus to explore the perceived feasibility and optimal design of Tobacco Cessation on Prescription (TCP) in PHC, targeting disadvantaged groups in Sweden.

**Methods:**

This qualitative study is based on semi-structured interviews with 32 participants including (1) three experts in lifestyle interventions on prescription, (2) 14 healthcare providers and (3) 15 clients from three PHC centres in socioeconomically disadvantaged areas in Stockholm where tobacco use is high. The interviews were audio-recorded and transcribed verbatim. The manifest content of the transcripts was analysed according to a modified conventional approach to content analysis.

**Results:**

The interviewees proposed that TCP should include a template comprising the client’s information, evidence-based tobacco cessation options and choices for follow-up. They also suggested including information about the benefits of tobacco cessation, as well as empowerment and planning support tools. The participants also commented that other measures for tobacco cessation could be included on the prescription. From the clients’ point of view, the perceived advantages of TCP were often linked to an emotional meaning (e.g. increased motivation to quit tobacco use, sign of support from the healthcare system to seek care for tobacco cessation). For providers, advantages with TCP were frequently related to a practical meaning (e.g. improved documentation and facilitation of tobacco cessation treatment). The disadvantages identified were mainly connected with the future implementation of TCP (e.g. low self-efficacy among clients and providers).

**Conclusions:**

TCP was perceived to be a useful tool for both clients and providers, potentially facilitating a structured and effective approach to tobacco cessation in PHC, and targeting disadvantaged groups. More research is needed to develop the prescription and investigate its effectiveness and cost-effectiveness compared to current strategies for tobacco cessation in a PHC setting.

## Background

Tobacco use is the leading preventable cause of death worldwide [1], estimated to cause approximately 10 % of all premature deaths in Sweden [2]. Furthermore, chronic illnesses caused by tobacco use remain a public health problem in Sweden—particularly in socioeconomically disadvantaged groups, where tobacco use is markedly higher than in the general population [3]. Since tobacco cessation has been found to reduce the risk of tobacco related morbidity and mortality [4], it is a prioritised target area in Swedish public health policy [5]. How tobacco cessation should be tackled is described in the National Board of Health and Welfare’s guidelines for disease prevention methods [6]. Recommended treatment options include different types of counselling (simple advice, counselling, qualified advice, web- and computer-based counselling and proactive telephone counselling) with or without pharmacotherapy (nicotine replacement therapy, varenicline and bupropion) [6]. However, these types of health-promoting interventions often fail to reach those in greatest need [6]. Difficulties in reaching disadvantaged groups with tobacco cessation interventions could be explained by a lack of motivation, low self-efficacy, reduced social support for quitting and understanding of the harmful effects of tobacco use, a stronger addiction to tobacco, targeted marketing by the tobacco industry and low adherence to treatment [7]. In addition, the costs of pharmacotherapy and counselling present particular barriers for disadvantaged tobacco users to seek and complete treatment for tobacco cessation [8, 9].

Studies conducted in Stockholm on healthcare consumption in different social settings show that individuals with low socioeconomic status and foreign origin often visit primary healthcare (PHC) [10]. PHC may therefore be seen as a potential platform for reaching disadvantaged groups with health-promoting activities. Although the utilisation of PHC is on a par with other groups of the population, the proportion of medical needs not met is higher among disadvantaged groups who frequently feel discriminated against by the healthcare system [10]. Among PHC staff, there is a need for more knowledge and training in how to work with disadvantaged groups for efficient health promotion [11, 12]. There is also a need for healthcare programmes, routines and documentation tools to facilitate such efforts [13].

Physical activity on prescription (PAP) is a lifestyle intervention on prescription, which has been successfully tested and validated in the general population in Sweden [14, 15]. Methods to prescribe physical activity have also been applied in the healthcare systems in Norway, Denmark, Finland, Great Britain, New Zealand, Australia, USA and Spain [16–22]. In Sweden, PAP is adhered to in a way comparable to medical treatments of chronic diseases and has been found to lead to increased physical activity levels and improved health and quality of life [15]. Previous research suggests that there is a demand among tobacco users in Sweden for receiving tobacco cessation support from healthcare providers [13], e.g. through referrals [23]. Therefore, Tobacco Cessation on Prescription (TCP) has the potential to become a comprehensive, health-promoting tool in the Swedish PHC setting. TCP may also increase the willingness amongst clients to receive assistance, as tobacco cessation would obtain a status like other medical care [24]. A prescription approach to tobacco cessation treatment could also be relevant in other countries where prescriptions to promote lifestyle changes are already in use. This study aims to explore the perceived feasibility and optimal design of TCP as an innovative and complementary tool for tobacco cessation in PHC, targeting socioeconomically disadvantaged groups in Sweden.

## Methods

### Recruitment and sample selection

An exploratory study design, based on semi-structured interviews, was adopted to gather an in-depth understanding of the informants’ views on TCP and related issues. This approach was chosen since it is recommended when there is little knowledge about the subject of interest [25]. Experts in lifestyle interventions on prescription, adult tobacco users and PHC providers were purposively sampled [26] and invited to participate. Purposive sampling was applied since it is recommended in qualitative studies where the researchers are interested in interviewing those with the best knowledge concerning the subject of interest [27]. Experts were identified through their involvement in previous research projects conducted in Sweden on lifestyle interventions by prescription and were recruited to participate by the researchers. Clients and providers were recruited from three PHC centres situated in socioeconomically disadvantaged areas in Stockholm. The areas were identified through a socioeconomic index [28] that is used by the Stockholm County Council to follow up resource allocation in PHC and takes into account the income, educational level, health status and ethnicity of the population living in the area. Providers were licenced medical professionals who were identified and recruited by the researchers; clients were identified and recruited by the PHC staff. Eligible clients were self-reported tobacco users over 18 years of age who visited the PHC centres that were involved in the study. As an incentive, the participants received a 100 SEK gift card for partaking in the study. Of the approximately 50 participants that were invited to participate, 32 were interviewed, of which three were experts, 15 were clients and 14 were providers. Further details on the participant characteristics are presented in Table 1. Most non-participants were clients, representing different sexes and ages. Reported reasons for non-participation were lack of time and interest.

**Table 1:** Participant characteristics

Characteristic	Total N (%)	Respondent category		
		Client	Provider	Expert
		% (*n* = 15)	% (*n* = 14)	% (*n* = 3)
Age				
18–29	2 (6)	7	7	0
30–39	4 (13)	13	14	0
40–49	9 (28)	27	21.5	67
50–59	9 (28)	33	21.5	33
60–69	8 (25)	20	36	0
Sex				
Female	20 (62.5)	40	93	33
Male	12 (37.5)	60	7	67

### Data collection

Data was collected between February and May 2014 through semi-structured interviews in conversational form, based on interview guides with open-ended questions, developed specifically for each respondent category (Appendices 1, 2, 3). Clients and providers were asked about their tobacco use, experiences of tobacco cessation and opinions on the concept of, and requested content on, TCP. It was described as a prescription form similar to prescriptions of pharmaceuticals and physical activity, which could be administered by a healthcare provider to a client in the PHC setting to support and facilitate tobacco cessation. The participating experts, providers and clients were all previously familiar with prescriptions of pharmaceuticals. All of the experts and providers, and most of the clients, were also previously familiar with PAP. Clients were asked about their health. Experts were asked about lessons learned from other lifestyle interventions on prescription, the possibilities and barriers associated with these interventions, and how they might impact upon TCP. Examples of interview questions related to the aim of the study were: *“If you could prescribe/get a prescription for tobacco cessation, what would you think about that?”* and *“What are the possible advantages and disadvantages of TCP?”*

Twenty-eight of the interviews were conducted face-to-face, while two of the expert interviews and two of the client interviews were conducted via telephone based on participant preference. The face-to-face interviews were conducted in private rooms, located in the PHC centres where the participants were recruited. Due to specific requests from participants, one client interview was conducted in the waiting room and one provider interview was conducted in the interviewer’s office. On average, the interviews lasted for approximately 30 min. All interviews were conducted by an experienced interviewer with degrees in health communication and global health science. All but one of the interviews were conducted in Swedish. The exception was carried out in Arabic in collaboration with a translator. All interviews were audio-recorded and supplemented with field notes. The interviewer transcribed each interview verbatim. A professional agency transcribed and translated the interview from Arabic to Swedish.

### Data analysis

Data was collected until a point in time at which the analysis provided no more emergent patterns [29]. The final analysis was based on a conventional approach to content analysis [30]. The chosen approach was considered appropriate since it is recommended in studies when existing theory or research literature on the phenomenon that is being explored is limited [30]. In order to obtain an overview of data, the transcripts were read several times by two members of the research team. The transcribed text on TCP related issues was extracted from the responses of all three categories of respondents and was brought together to constitute the unit of analysis. The responses from all participants were analysed together to value each respondent category’s opinions equally and to get an overview of the overall opinions on TCP from all included stakeholders. However, their accounts are reported separately in the results to highlight similarities and differences between the responses from different categories. Meaning units were identified, abstracted inductively and labelled with codes after analysing the text word by word. Based upon the manifest content of the text and differences and similarities between the codes, these were combined into thirteen sub-categories and four categories. With the exception of condensing meaning units and formulating themes, the analysis was conducted as described by Graneheim and Lundman [31]. The identified codes, sub-categories and categories are presented in Table 2. The software QSR NVivo 10 was used in the coding process. The credibility of the analysis was enhanced by random checks of the analysis, performed by another member of the research team and an independent researcher experienced in qualitative methods but not part of the research team. The interrater reliability was not formally measured but was perceived to be high by the coders. Contradicting opinions between the coders were discussed until a final categorisation of the codes was agreed upon. Results of the analysis were also checked for credibility of interpretation with three providers and two clients. Selected quotations are presented to reflect common answers from the respondents. Differences in responses are also highlighted.

**Table 2:** Identified codes, sub-categories and categories

Code	Sub-category	Category
Cessation activity, general content, empowerment, contact, medical content, planning support, referral	Content	The prescription
Mode of administration, layout	Design	
Prescription for all smokers, prescription for those with a health problem, prescription for those with high self-efficacy	Target group	Usage
Prescription whenever, prescription as soon as possible, prescription at a certain point in time	When to receive TCP	
No follow-up, when to follow-up, how to follow-up, why follow-up	Follow-up	
Shared responsibility, manager responsibility, occupational group responsibility	Responsibility	
Guideline characteristics, guideline content	Guidelines	
Positive emotional meaning, positive practical meaning, positive characteristics	Advantages	Expected results
Negative emotional meaning, negative practical meaning	Disadvantages	
Providers’ self-efficacy, clients’ self-efficacy	Adherence	
Others indifferent, others positive, others negative, others ambiguous	Perceptions of others^a^	
Implementation prerequisites, person-centeredness, TCP as a package	Implementation	Feasibility
Motivation and competence, budget, organisational obstacles, infrastructure, time, capacity building	Organisational aspects	

^a^From a client’s perspective “Perceptions of others” refer to perceptions of family members, friends and society. From a provider’s perspective it refers to perceptions of patients and colleagues

### Ethics

Ethical approval was obtained from the Regional Ethical Review Board in Stockholm in the autumn of 2013 [no: 2013/2264-32/2]. Written informed consent was obtained from the participants before the start of each face-to-face interview and verbal informed consent was obtained before the start of each telephone interview. The anonymity and confidentiality of the participants was ensured by coding the participants as numbers (1–32) and removing all identifiers but the respondent category in the presentation of the results. Furthermore, best practice guidelines for qualitative research [32] were applied to ensure quality.

## Results

### The prescription

#### Content

Regarding the content of the prescription, providers suggested that TCP could include information about the client’s diagnosis and contact person at the local PHC centre, as well as the type and time of follow-up. Furthermore, both providers and clients stated that TCP could contain a list of different evidence-based cessation options, such as medications (varenicline, bupropion), aids (e.g. nicotine patches or gum replacement therapy), and physical activity/PAP.

*“There should be different aids […] and pharmaceuticals [to choose from] on the prescription itself.”* (Provider 18).

*“There has to be a pre*-*printed list of options [for tobacco cessation] that there are to get help from […] so that one can check [a box] and write maybe freely the treatment plan and how one plans to follow it up.”* (Provider 7).

Moreover, presenting alternatives for counselling on the TCP was suggested, e.g. via telephone or by referral to the Swedish quitline. Referrals to support groups, tobacco cessation courses and other relevant units or lifestyle clinics were also mentioned. Information about the health benefits of tobacco cessation and cessation planning support, e.g. a reduction scheme or an action plan, were also requested by providers to be part of the prescription. Additionally, providers depicted TCP as comprising a list of empowerment options. For example, some providers described a “*reward system*”, allowing clients to reward themselves after not having used tobacco for a certain amount of time, or after having saved a certain amount of money by no longer purchasing tobacco products.

“*I usually suggest [the client] going on a trip, to have a goal, or that they can go out and enjoy a good meal [as a reward for tobacco cessation].*” (Provider 12).

Clients, providers and experts also suggested other non-evidence based cessation support ideas, such as relaxation, laser therapy, eating fruit, drinking water, engaging in cultural, outdoor and other “*fun*” activities, holidays, church visits, use of self-help books and e-cigarettes to be included on the prescription.

*“I think it’s good to have some kind of physical activity [on the prescription] but also to have some kind of relaxation [technique] […] like mindfulness or medical yoga that ensures that you relax […] and explain that ‘You are going to experience abstinence but if you hold out [relax] for a while it will also disappear´.”* (Expert 31).

#### Design

Some providers compared the design of TCP with that of PAP. Furthermore, the prescription was suggested to look “*visually nice*”. Most providers and clients described TCP as a paper prescription, as opposed to an electronic prescription.

“*[TCP should be] like a real [paper] prescription. Anything else would be worthless because then you wouldn’t feel the same dignity as you feel with a doctor.*” (Provider 10).

*“Nowadays, all prescriptions for medications are electronic […]. But at the same time it would be weird if you had an e*-*prescription if it was not for medications, if it was an e*-*prescription for some kind of activity one should do.”* (Client 21).

### Usage

#### Target group

Six of the providers stated that they would prescribe TCP to all tobacco users and nine of the providers stated that they would mainly prescribe it to tobacco users with smoking-related health problems like asthma/COPD, cardiovascular diseases, diabetes, high blood pressure, high cholesterol or allergies.

*“I don’t think anyone should smoke! But those with diabetes, cardiovascular problems, asthma/COPD are the largest [target] groups [for TCP]. But again, it can be [prescribed] to all [tobacco users].”* (Provider 4).

#### When to receive TCP

Clients frequently responded that they would like to be prescribed TCP “*whenever*”, “*as soon as possible*”, or at a certain point in time, e.g. once health status worsens, improves, or once the decision to quit tobacco use has been made.

#### Follow-up

Providers considered a follow-up of the prescription as important. It was also considered significant for clients to keep track, be controlled, see progress, get motivation and find alternative solutions in case the prescribed cessation option did not work. However, some clients wished not to be followed up.

“*I think it [follow*-*up] is needed. So that they [providers] hear how it went […]. That is always the best. [To] keep track.*” (Client 14).

The clients suggested different time intervals for the follow-up; weekly, monthly, continuously, or sporadically.

*“Maybe in the beginning [the client could be followed*-*up] each week, if possible […] and later a bit more sporadically.”* (Client 20).

It was suggested by two clients and two providers that the follow-up could be carried out via telephone. One provider commented that the follow-up by telephone could be done by a physician while another provider proposed that it could be done by the Swedish quitline. In addition to the cessation progress and how TCP worked, it was suggested by a client that certain body functions could also be tested during the follow-up.

#### Responsibility

Providers suggested different ways of distributing the responsibility for TCP and its implementation—from being a shared responsibility between *“everyone”*, to being an exclusive responsibility of managers and specific occupational groups, such as physicians, nurses, occupational therapists, physiotherapists, dieticians or specific units like asthma/COPD clinics.

*“I think [it should be] like with PAP, that all nurses, all doctors, all [healthcare providers] who have contact with the patient, can offer [TCP], except for pharmaceuticals*–*that is the doctors responsibility […] I don’t think it [prescription of TCP] should be restricted to a certain [occupational] category. Then the usage would be lower.”* (Provider 18).

#### Guidelines

Several providers requested guidelines for the prescription of TCP, including information about recommended treatment and dosage alternatives and practical steps for healthcare providers to take in order to best support their clients in quitting their tobacco use.

*“There could be some kind of [recommended] chain [of events, like] ´Write a prescription´, ´Inform […] about what happens when you smoke´, ´Inform about the Swedish quitline´ in different steps and if that’s not enough´ Refer to a lifestyle clinic’ as the next step.”* (Provider 3).

Moreover, the providers expressed that the guidelines should be easy to use, permissive, generous, structured, and adaptable, without judgement and encouraging the provider to always take up the issue of tobacco use.

### Expected results

#### Advantages

TCP was often related to a positive emotional meaning for the clients. Participants from all three respondent categories expressed that TCP could give the client motivation or help with cessation, and serve as a proof/confirmation for the seriousness of tobacco use as a health threat. In addition, some clients and providers perceived TCP as an opportunity, a positive pressure, a first step, a reminder, an inspiration and a guarantee for successful cessation.

*“If one gets a’push’, a bit of support, then it would make it easier, at least for me [to quit smoking]. […] I would have made up my mind, after having talked to another person about this [tobacco cessation] and gotten a medication [on prescription]. Then I have to put the work in, make sure that it happens”.* (Client 20).

In contrast to clients, providers often linked the advantages of TCP to a practical meaning. TCP was seen as a useful tool in the daily routine and the follow-up of tobacco cessation treatment. It was also seen as an advantage that all healthcare personnel would follow the same scheme in tobacco cessation. Providers considered TCP as being valuable for documentation, planning purposes, data collection and as an information source.

“*It [tobacco cessation] becomes clearer with this method [TCP] and then patients can choose themselves which method they want to choose […]* – *it becomes much easier for healthcare personnel.*” (Provider 18).

Providers often described TCP’s character as concrete, “*official*” and structured. In addition, TCP was seen as a tool for both clients and providers. Several clients and providers contended that the existence of TCP would show support from the health system and the healthcare staff in tobacco cessation.

*“And he [the patient] can say ‘Look, I got a prescription because the healthcare system says that I should stop smoking’, then they [the patients] can get motivation from their [family/friends], ‘God, he got a prescription. Now he shouldn’t smoke’ or ‘[…] it is serious, so now we must make sure that he does that [stop his tobacco use]’.”* (Provider 8).

“*Then [with TCP] I know that I have the right to seek [care for tobacco cessation].*” (Client 20).

#### Disadvantages

Disadvantages of TCP were frequently related to an emotional meaning for the participants. For instance, a lack of self-efficacy was seen as a disadvantage by some of the clients.

*“The disadvantage would be that I’m not strong enough to accomplish that [tobacco cessation with TCP].”* (Client 20).

Furthermore, some providers perceived TCP as potentially pressuring the client in a negative way, or prescription of TCP as a “*stressful*”, “*humiliating*” or “*ridiculous*” experience.

*“He [the client] is allowed to buy Nicotine Replacement Therapies (NRTs) […] without having this paper [TCP] in his hands. That [getting a prescription for tobacco cessation] I can imagine some might think is a little humiliating”.* (Provider 5).

In terms of practical meaning, providers related disadvantages of TCP to the risk of “*prescribing wrongly*” (i.e. prescribing something that would not help the client to quit) and scepticism towards TCP’s accessibility language-wise for clients with insufficient Swedish language skills. Another disadvantage perceived by providers, was “*the risk that TCP would be like PAP*” in a negative way, i.e. “*complicated*” in terms of finding appropriate institutions to refer the client to, and “*tricky*” when it comes to forms and how to fill them out. Other disadvantages mentioned by providers related to insecurities about TCP’s content, lack of evidence for the prescription approach, potential technical problems with integrating the prescription into the electronic medical record, and a perceived lack of time and human resources for prescribing TCP.

“*There is the disadvantage of having ‘one additional thing’ [to do].”* (Provider 18).

The risk of forgetting, or not following up TCP, was also mentioned as a disadvantage. Some clients were insecure about TCP and some providers found it unnecessary, or perceived it as a way of “*labelling smoking as a disease*”.

#### Adherence

Experts and providers expected the adherence to TCP to be lower among healthcare providers and clients with low levels of self-efficacy. None of the clients discussed adherence during their interviews.

*“I think it [the adherence to TCP] would be the same as with all other tobacco cessation [interventions]. I think it is more about motivating them [the clients]. Maybe the prescription could get them more motivated and then the adherence would be higher.”* (Provider 11).

#### Perceptions of others

Clients and providers anticipated others not to care about TCP, being unsure (surprised or sceptical), perceiving it negatively (against TCP, frustrated, afraid of tobacco cessation), positively or *“worth trying”*.

*“I think that there are both, some who will perceive it [TCP] as positive and some that will question it.”* (Provider 5).

### Feasibility

#### Implementation

In order to facilitate the implementation of TCP, some providers suggested that it would be beneficial if TCP was designed as a “*package*”, linked to the electronic medical record, counselling and additional information. It was also considered important that the prescription was adaptable to the individual—even language-wise. One provider mentioned that a link between TCP and relevant collaborators could be beneficial. In order for TCP to be successful, some experts, clients and providers considered it necessary for the provider to ask the client how he/she would like to go about tobacco cessation, which goals the client would have in terms of cessation and what the client would like to do to compensate for his/her tobacco use, before deciding together which of the available cessation options to choose. It was seen as a tool that could promote shared decision making and to more actively involve the client in the choice of cessation treatment.

*“If I decide that’Ah, this [cessation option] suits you’, the patient won’t buy it. If you have it [the cessation options] on the prescription, then you can go through [them] with the patient. ‘This is a nicotine patch. Have you tried? Or what do you think, is gum better for you?’ Then you could tick [choose] the [type of] aid together.”* (Provider 18).

Other mentioned prerequisites for a successful implementation were that TCP should be simple, structured, easily accessible and usable.

*“The prescription has to be found in Take Care [the electronic medical record] or the data system […] so that I can pick it up easily. It [the form] must be very easily filled in.”* (Provider 18).

#### Organisational aspects

From the provider’s points of view, TCP was suggested to require motivation and competence from the healthcare personnel, but also an apposite budget, infrastructure and teamwork. Time was mentioned as an important factor. Some providers described a lack of time for prescribing TCP, while others could imagine implementing TCP in their daily routine. Some providers also stated that the County Council (healthcare system owner and main provider) would be responsible for allowing more time for prescribing TCP.

*“As it is now […] we wouldn’t have time [to prescribe TCP] because we [healthcare providers] don’t have enough expertise and it [TCP] requires a lot more. You don’t just give them the note [prescription], so to speak.”* (Provider 2).

Some experts and providers perceived difficulties reaching the intended target group. Moreover, some providers perceived a lack of evidence concerning the prescription approach. Some providers also found it difficult to introduce new methods in PHC. Experts and providers stated that it would be beneficial if clients as well as providers received an economic advantage from using TCP. Some clients also expected an economic benefit for receiving TCP.

*“‘On prescription’, then it [the treatment] should be included in the reimbursement scheme. Otherwise it [tobacco cessation treatment] is pretty expensive stuff, I have heard.”* (Client 22).

Furthermore, a good introduction to TCP for healthcare personnel was found important and expected to have a positive effect on its uptake.

## Discussion

Overall, the concept of TCP was considered a valuable support for both providers and clients, facilitating tobacco cessation treatment among disadvantaged groups in PHC in a new, easy and structured way. The prescription, containing an overview of available cessation options and planning support, could serve as an information source, but also as a basis for discussion between the provider and the client. TCP would allow each client to—together with their caregiver—choose the cessation options that suit them the best, tailoring the prescription to meet their individual preferences and needs. Previous research suggests that personalised and non-judgemental approaches [8] that combine behavioural support and pharmacotherapy are effective in supporting and engaging disadvantaged tobacco users to quit [33].

The importance of offering and combining a variety of treatment options in tobacco cessation became clear in the study, since several participants were sceptical towards the use of pharmaceuticals but positive towards some non-traditional measures for tobacco cessation. A recent clinical review suggests that some of the methods mentioned by the participants (e.g. relaxation techniques, drinking water, eating low fat foods like fruit and engaging in physical, fun and health promoting activities) may be used as strategies for stress management or distraction to reduce cravings and withdrawal symptoms [34]. Although these options are unlikely to be pre-printed on the prescription, these could beneficially be discussed in relation to the prescription of TCP. If the client shows interest in a non-evidence based and potentially harmful option in these discussions, such as e-cigarettes, this should not be promoted. Self-efficacy could be improved by involving clients in the choice of treatment and by providing empowerment and planning support on the prescription. The importance of self-efficacy in tobacco cessation has previously been highlighted in the literature [35–37]. It was also considered the most influential determinant of successful cessation among providers and clients in the study.

Although this was not the focus of the study, a need for increased awareness about available cessation alternatives emerged, since few of the participants seemed to know about the services available to help them. Similar findings have been presented in previous research, which also suggests that there are misconceptions in this population about the availability and effectiveness of such services [8]. In order to reach disadvantaged groups with tobacco cessation interventions it is recommended that free or heavily subsidised NRTs should be offered and that targeted marketing strategies using mass media should be applied [38]. Currently, neither of these strategies are used in Sweden. Although the long term effects of these strategies have not been established [33, 39–41], informing the target group about TCP including its potential financial benefits could increase the demand and utilisation of NRTs and other tobacco cessation treatments among disadvantaged tobacco users in PHC in Sweden. The fact that economic incentives influence an individual’s willingness to participate in other life style intervention on prescription programmes support this statement [42].

Perceived challenges with the implementation of TCP were related to a lack of time, resources and knowledge to prescribe TCP. The same obstacles and the need for education, routines and guidelines have previously been stressed by primary care providers in relation to physical activity counselling [43] and prescriptions [44]. Development of guidelines and education of PHC staff in how to prescribe TCP should therefore be considered crucial in the implementation process. Since the lack of reimbursement for preventive counselling is also considered a major barrier [43], financial incentives may be needed to motivate PHC staff to prescribe TCP. Previous research suggests that financial incentives enhance the likelihood that PHC will devote time to health promotion [45]. Without financial support, there is a risk that other activities that are reimbursed will be prioritised [46]. Despite these challenges, the prescription approach has previously been found effective in changing lifestyle behaviour when clear advice was given and prescribed with the same conviction as a drug [15]. Studies have shown that PAP increases self-efficacy [47] and physical activity, while decreasing the proportion of inactive individuals and reducing costs for inactivity by 22 % [48]. The adherence to PAP has further been found to be similar to that of other treatments for chronic diseases [15]. Although the adherence to prescribed medications is generally lower in disadvantaged groups [8], the study participants reported that they would become motivated by receiving a prescription on tobacco cessation. Most of the participants also stated that they had high levels of trust in their caregivers and considered TCP to be a demonstration of support from the healthcare system. Since a client’s trust in the provider and the healthcare system has a positive effect on adherence to treatments [49–51], TCP could be an effective approach in achieving tobacco cessation among disadvantaged tobacco users in Sweden.

Introducing TCP as an intervention model for tobacco cessation could be a means for implementing the national guidelines for disease prevention and supporting PHC to integrate health promotion into its daily activities. Better support from PHC staff in tobacco cessation could lead to more successful quit attempts and health improvements in the population. TCP may also help to reduce health inequalities by targeting disadvantaged groups who have a higher prevalence of tobacco use and related health problems compared to the general population. However, before TCP can be implemented, more research is needed to further develop the content and the design of such a prescription. The effectiveness and cost-effectiveness of TCP should also be evaluated in comparison with current tobacco cessation strategies to determine whether or not its implementation would be an efficient allocation of society’s limited resources. Such an intervention study is currently under development.

A possible limitation to the study was that most of the interviews were conducted in Swedish when neither the interviewer, nor some of the participants were native Swedish speakers. A language barrier may thus have been introduced in the communication between the interviewer and the respondents, as well as in the interpretation of data [52]. However, the understanding was enhanced through enquiries, audio-recording of the interviews and validation of the materials by a native Swedish speaker. In addition, social and cultural differences between the interviewer and the participants (e.g. socioeconomic status, nationality, age, gender) may have influenced the data collected [52]. The public health background of the researchers performing the analysis may also have influenced the manner in which the material was coded and categorised. Another limitation of the study was that only the manifest content of the interview transcripts was considered in the analysis. This is a disadvantage of using a conventional approach to content analysis. However, this approach can be useful in concept development or model building [53] which was closely related to the aim of this study and therefore considered appropriate. Further, it should be noted that the findings from this study are based on the views of 32 participants. Although the credibility of the findings was enhanced by including a diverse sample of participants, representing different ages, sexes and respondent categories, there was only one male healthcare provider in the sample. Overall, fewer male providers were asked whether they wanted to participate, as predominantly female nurses were, or felt like they were, responsible for tobacco cessation at the PHC centres that were involved in the study. A prescription approach to tobacco cessation treatment could be relevant in other countries as well, for example where physical activity or other lifestyle interventions are already being prescribed. However, the approach would have to be modified to the context in which it is intended to be used.

## Conclusions

A TCP tool was perceived to be useful for both clients and providers, potentially facilitating a structured and effective approach to tobacco cessation in PHC, targeting disadvantaged groups. More research is needed to develop the prescription and investigate its effectiveness and cost-effectiveness compared to current strategies for tobacco cessation in a PHC setting.

## Appendix 1: Interview guide for experts

1. What is [Lifestyle intervention X] on prescription?
  - How was it developed?
  - What is its purpose? Who is the target group?
  - What does the prescription look like?/What does it contain?
    - Why? (How was the content determined?)
    - What scientific evidence is the content based on?
  - Who is involved in the prescription process?
    - How motivated are those in prescribing [Lifestyle intervention X] on prescription?
  - Are we talking about a paper or electronic prescription?
    - What effect do you think that the mode of administration has?

2. What are the enabling factors for [Lifestyle intervention X] on prescription to work as intended?
3. What are the barriers for [Lifestyle intervention X] on prescription to work as intended?
  - Motivation?
    - Prescribers
    - Clients
4. What have you learned from your experiences with [Lifestyle intervention X] on prescription?
5. What do you think about the suggested Tobacco Cessation on Prescription?
  - Do you think it would have an added value for tobacco cessation?
  - Do you think it would be helpful in reaching disadvantaged groups?
6. Do you have any further comments?

## Appendix 2: Interview guide for clients

### Personal details

1. How would you describe your health in relation to others of the same age?

### Tobacco use

2. Describe your tobacco use (what, for how long, how much, when)
3. How do you feel about quitting?
  - Why? (motivation)
  - Do you have a strategy/plan for how to quit?
4. What support/help do you need in order to quit?

### Experiences with tobacco cessation

5. Have you previously tried to quit?
  - How? Why?
  - What challenges did you experience during your quit attempt(s)? (e.g. weight gain)
6. What do you know about available support for tobacco cessation (here in Stockholm)? Give examples.
7. Please describe any support you have sought to quit.
8. What experiences does your friends and family have from tobacco cessation that you know of? Results?

### Tobacco Cessation on Prescription

Tobacco Cessation on Prescription means that your doctor/nurse/physiotherapist or other healthcare provider could prescribe you *something* to support you in quitting your tobacco use.

9. If you could get a prescription for tobacco cessation…
  - What would you think about that?
  - What would the prescription say? Why?
  - What would it look like? Why?
10. Please describe how you think Tobacco Cessation on Prescription could support someone to quit their tobacco use.
  - Why? Why not?
  - What are the possible advantages of Tobacco Cessation on Prescription?
  - What are the possible disadvantages of Tobacco Cessation on Prescription?
  - How do you think others would perceive Tobacco Cessation on Prescription? (attitudes)
11. When would you like healthcare providers to prescribe tobacco cessation to you?
12. How would you like healthcare providers to follow-up the prescription?
  - How often?

### Further comments

13. Do you have anything else you would like to add?
14. How did you perceive this interview?

## Appendix 3: Interview guide for providers

### Personal details

1. What is your role at this primary healthcare centre?
  - What is your highest education?
  - How long have you been working in primary care in Stockholm/this primary healthcare centre?

### Tobacco use

2. Please describe your experiences and the experiences of friends and family with regard to tobacco use.
  - Did you/they wish to quit using tobacco? Why? How?

### Experiences with tobacco cessation

3. What are your experiences with tobacco use and tobacco cessation among your patients?
  - Do you offer your patients support for tobacco cessation or do your patients ask you for support to quit their tobacco use?
  - How do you support them?
  - How do you think you could support your patients better?
  - What do you need to better support patients to quit? Give examples.
4. Please describe how you perceive their knowledge about tobacco cessation.
5. What support for tobacco cessation (here in Stockholm) do patients know of?

### Tobacco Cessation on Prescription

Tobacco Cessation on Prescription means that you and other healthcare providers could prescribe *something* to your patients to support them to quit their tobacco use.

6. If you could prescribe tobacco cessation…
  - What would you think about that?
  - What would the prescription say? Why?
  - What would it look like? Why?
  - What would guidelines for the prescription look like? Why?
7. Please describe how you think Tobacco Cessation on Prescription could support patients to quit their tobacco use.
  - Why? Why not?
  - What are the possible advantages of Tobacco Cessation on Prescription?
  - What are the possible disadvantages of Tobacco Cessation on Prescription?
  - How do you think patients would perceive Tobacco Cessation on Prescription? (attitudes)
8. Please describe how you think Tobacco Cessation on Prescription could help healthcare providers to support patients to quit their tobacco use.
  - Why? Why not?
  - What are the possible advantages of Tobacco Cessation on Prescription?
  - What are the possible disadvantages of Tobacco Cessation on Prescription?
  - How do you think your colleagues would perceive Tobacco Cessation on Prescription? (attitudes)
  - What time, budget and infrastructure would be needed to implement Tobacco Cessation on Prescription?
9. Who would you prescribe tobacco cessation to?
10. Please describe what you expect the compliance to Tobacco Cessation on Prescription would be.
11. Who do you think should be responsible for prescribing Tobacco Cessation on Prescription? Give examples.

### Further comments

12. Do you have anything else you would like to add?
13. How did you perceive this interview?
